# In memoriam of Prof. Alessandro Zuddas (1957–2022)

**DOI:** 10.1186/s13034-022-00504-8

**Published:** 2022-08-18

**Authors:** Tobias Banaschewski, Philippe Auby

**Affiliations:** 1 Department of Child and Adolescent Psychiatry and Psychotherapy, Central Institute of Mental Health, Medical Faculty Mannheim, Heidelberg University, Mannheim, Germany; 2 University Department of Child and Adolescent Psychiatry, Children’s Hospitals of Nice CHU-Lenval, Nice, France; 3 CoBTek, University of Côte d’Azur, Nice, France

Sadly, Prof. Alessandro Zuddas, a much respected and loved colleague and teacher, died unexpectedly on 9 July 2022. He was a Professor of Child Neuropsychiatry at the University of Cagliari (Italy) in the Department of Biomedical Sciences, Section of Neuroscience and Clinical Pharmacology, Director of the Child and Adolescent Neuropsychiatry Unit at the “G. Brotzu” University Hospital (Cagliari), and Coordinator of the Resident Programme in Child Neuropsychiatry at University of Cagliari.

Alessandro had a deep knowledge of both basic and clinical neuroscience, having completed residences in neurology, pharmacology, and child neuropsychiatry, followed by training in basic neuroscience as a Fogarty Fellow at the National Institute of Neurological Disorders and Stroke (NINDS-NIH, Clinical Neuroscience Branch, Bethesda MD, USA). His career focused on child psychiatry, mostly neurodevelopmental disorders, especially Attention-Deficit/Hyperactivity Disorder (ADHD). He has contributed significantly to the advance and development of European Child and Adolescent Psychiatry. Alessandro was the principal investigator in multiple European Union-funded research projects and on the forefront of research and advocacy for new psychopharmacological approaches to treating children with mental disorders. He was an extraordinary active member of Italian and European scientific societies, serving on the boards of the Italian Society of Child and Adolescent Neuropsychiatry (SINPIA) and the Italian Society of Neuropsychopharmacology (SINPF) and was appointed as an expert in Child Neuropsychiatry and ADHD by the Italian Health Ministry. Alessandro was a highly important and active member of the European Network on Hyperkinetic Disorders (Eunethdis) Network, the Eunethydis ADHD Guideline Group (EAGG), the European College of Neuropsychopharmacology (ECNP), acting as Chair of the Child and Adolescent Neuropsychopharmacology Network and Coordinator of the ECNP School of Child and Adolescent Neuropsychopharmacology. He was also a member of the Editorial Board of several journals, being co-Editor of European Child and Adolescent Psychiatry between 2005 and 2015. Alessandro also showed a true teaching dedication and was a role model and supporter of younger generations of child psychiatrists. This was the case not only in Italy but also across all of Europe through the ECNP School of Child and Adolescent Neuropsychopharmacology, which he promoted and advanced over the years with exceptional leadership to turn it into an outstanding learning opportunity for a large number of young psychiatrists.

Most importantly, Alessandro was an extremely gentle, generous and cooperative person and mentor, able to create a productive and pleasant environment. He has been enthusiastic, charismatic and passionate, kind and honest, as well as a wise and charming person. We are grateful to have had the opportunity to know him as a colleague, mentor and friend. He was truly extraordinary and irreplaceable. Prof. Alessandro Zuddas will be deeply missed and will always be remembered.

